# Hepatopathy Associated With Type 1 Diabetes: Distinguishing Non-alcoholic Fatty Liver Disease From Glycogenic Hepatopathy

**DOI:** 10.3389/fphar.2021.768576

**Published:** 2021-10-25

**Authors:** Jonathan Mertens, Christophe De Block, Maarten Spinhoven, Ann Driessen, Sven M. Francque, Wilhelmus J. Kwanten

**Affiliations:** ^1^ Department of Gastroenterology and Hepatology, Antwerp University Hospital, Edegem, Belgium; ^2^ Department of Endocrinology, Diabetology and Metabolism, Antwerp University Hospital, Edegem, Belgium; ^3^ Laboratory of Experimental Medicine and Pediatrics, University of Antwerp, Wilrijk, Belgium; ^4^ Department of Radiology, Antwerp University Hospital, Edegem, Belgium; ^5^ Department of Pathology, Antwerp University Hospital, Antwerp, Belgium; ^6^ CORE, Faculty of Medicine and Health Sciences, University of Antwerp, Wilrijk, Belgium

**Keywords:** type 1 diabetes mellitus, non-alcoholic fatty liver disease, metabolic dysfunction-associated fatty liver disease, non-alcoholic steatohepatitis, glycogenic hepatopathy

## Abstract

Autoimmune destruction of pancreatic β-cells results in the permanent loss of insulin production in type 1 diabetes (T1D). The daily necessity to inject exogenous insulin to treat hyperglycemia leads to a relative portal vein insulin deficiency and potentiates hypoglycemia which can induce weight gain, while daily fluctuations of blood sugar levels affect the hepatic glycogen storage and overall metabolic control. These, among others, fundamental characteristics of T1D are associated with the development of two distinct, but in part clinically similar hepatopathies, namely non-alcoholic fatty liver disease (NAFLD) and glycogen hepatopathy (GlyH). Recent studies suggest that NAFLD may be increasingly common in T1D because more people with T1D present with overweight and/or obesity, linked to the metabolic syndrome. GlyH is a rare but underdiagnosed complication hallmarked by extremely brittle metabolic control in, often young, individuals with T1D. Both hepatopathies share clinical similarities, troubling both diagnosis and differentiation. Since NAFLD is increasingly associated with cardiovascular and chronic kidney disease, whereas GlyH is considered self-limiting, awareness and differentiation between both condition is important in clinical care. The exact pathogenesis of both hepatopathies remains obscure, hence licensed pharmaceutical therapy is lacking and general awareness amongst physicians is low. This article aims to review the factors potentially contributing to fatty liver disease or glycogen storage disruption in T1D. It ends with a proposal for clinicians to approach patients with T1D and potential hepatopathy.

## Introduction

Type 1 diabetes mellitus (T1D) is caused by autoimmune destruction of the insulin-producing pancreatic β-cells resulting in chronic hyperglycemia and lifelong exogenous insulin dependency (American Diabetes Association, 2021a). T1D usually presents at a young age, contrasting type 2 diabetes (T2D), implying a long time spent with diabetes. Lifelong adherence to therapy is essential in T1D, which is difficult to obtain, certainly in adolescence (Datye et al., 2015). Adequate glycemic control is, however, important since fluctuations in blood glucose levels are predisposing factors for disturbance of glycogen homeostasis, as discussed later.

The pathogenesis of T1D is multifactorial (Paschou et al., 2018). The “accelerator” (Wilkin, 2001) and “overload” (Dahlquist, 2006) hypothesis postulates that β-cell stress caused by insulin resistance and increased insulin demand might contribute to T1D. Already in 1975, Baum et al. described that increased weight gain in infancy could be linked to the development of T1D (Baum et al., 1975; Lauria et al., 2015). This hypothesis is further fueled by an increased odds ratio to develop T1D when childhood adiposity was present (Censin et al., 2017), and a lower risk of T1D associated with the presence of an insulin sensitivity-increasing polymorphism (Raj et al., 2009). Since the global incidence of both (childhood) obesity (Blüher, 2019; Di Cesare et al., 2019) and T1D are rising (Mobasseri et al., 2020), there might be a common pathway leading to increased β-cell fragility and subsequent development of diabetes, in the presence of other stressors, leading to patients with both T1D and an already present chronic metabolic dysfunction (Liston et al., 2017).

Both T1D as T2D are strongly associated with multiple micro- and macrovascular complications (de Ferranti et al., 2014; Schofield et al., 2019). Mortality due to cardiovascular disease is, despite current treatment strategies, still increased in patients with T1D compared to the general population, with women being proportionally more affected than men (de Ferranti et al., 2014; Huxley et al., 2015; Khunti et al., 2015). A Korean nationwide study determined that mortality risk and cardiovascular disease is even higher in patients with T1D, as compared to patients with T2D, stressing the importance of cardiometabolic risk identification in these patients (Lee et al., 2019).

T1D-associated hepatic disease is less well known and documented, as is the case for T2D-associated hepatopathy. The most relevant chronic liver disease in this context is non-alcoholic fatty liver disease (NAFLD), characterized by large lipid droplet accumulation in hepatocytes in the absence of well-established causes of steatosis, e.g., the use of alcohol or steatogenic drugs (Marchesini et al., 2016; Chalasani et al., 2018). NAFLD usually, but not exclusively, develops in the context of metabolic disturbances such as overweight and diabetes (Godoy-Matos et al., 2020; Targher et al., 2021). The latest position statement of the American Diabetes Association (ADA) clearly mentions non-alcoholic fatty liver disease (NAFLD) as a common comorbidity of T2D and recommends screening all patients with T2D, elevated liver enzymes, and/or fatty liver disease present on ultrasound (American Diabetes Association, 2021b), which is in line with the joint European guideline (Marchesini et al., 2016). Neither the American nor the European guidelines mention NAFLD as a possible complication of T1D, nor provide any recommendation on how or whom to screen in T1D populations. Epidemiological data are limited, but according to the currently available data the prevalence of NAFLD in patients with T1D approximately equals that of the general population (about 20–25%), while T2D is reported to be more than 2-fold higher compared to patients with T1D (Younossi et al., 2016; Younossi et al., 2019a; de Vries et al., 2020). Nevertheless, a growing body of evidence suggests that individuals with T1D may also be at an increased risk of developing NAFLD, partly due to an increase in the presence of metabolic risk factors such as obesity and the metabolic syndrome in these patients, but also due to T1D-specific conditions that can propel metabolic dysfunction (Regnell and Lernmark, 2011; Barros et al., 2017; de Vries et al., 2020). Furthermore, disturbances in liver enzymes or imaging studies, suggesting underlying hepatopathy, have been commonly described in T1D (Leeds et al., 2009; Sherigar et al., 2018). Not all these cases are, however, attributable to NAFLD. The number of published case reports and series reporting glycogenic hepatopathy (GlyH), an entity characterized by excessive glycogen storage in hepatocytes leading to hepatomegaly and ultrasonographic findings similar to those of NAFLD, is rising as well, but estimates concerning the epidemiology of GlyH are absent (Sherigar et al., 2018). Therefore, this review aims to describe both the occurrence and pathophysiology of NAFLD and GlyH in T1D to raise clinical awareness and inspire further research. Furthermore, this review offers an overview of diagnostic modalities enabling clinical differentiation.

## Methodology

The authors performed a literature search of the most relevant articles in Pubmed (MEDLINE), Web of Science, and Google Scholar based on Medical Subject Headings (MeSH) terms and relevant synonyms. We searched using a combination of the following MeSH terms: “Type 1 diabetes” AND “NAFLD” or “Fatty Liver” or “Liver disease.” Further added to the search strategy were the words “Hepatic Glycogenosis” or “Glycogenic Hepatopathy” or “Liver Glycogenosis.” The reference lists of relevant articles were screened for additional search terms and articles. Abstracts were screened for relevance. All articles were restricted to English.

### Non-Alcoholic Fatty Liver Disease in Type 1 Diabetes: Epidemiology

NAFLD is highly prevalent in the world, with a quarter of the global population affected, and is clearly associated with the metabolic syndrome (Younossi et al., 2016; Younossi et al., 2019b). The prevalence is higher in individuals with T2D, with an estimated global prevalence of 55.5% (Younossi et al., 2019a). In comparison to epidemiological data on NAFLD in T2D, the number of studies addressing the prevalence in patients with T1D are limited.

A recent meta-analysis of twenty studies in 3,901 individuals with T1D showed that NAFLD is present in 19.3% of cases, increasing to 22.0% in adults only (de Vries et al., 2020). Interestingly, prevalence rates were highly discrepant when looking more in detail at the type of diagnostics. Indeed, ultrasound-based studies (*n* = 13) reported the highest prevalence rates (27.1%) compared to studies (*n* = 4) relying on magnetic resonance imaging (MRI) (8.6%) or the only available study evaluating NAFLD based on liver biopsy (19.3%) (de Vries et al., 2020).

A potential limitation of these included studies is that they are in all but one based on non-invasive techniques, thus only able to estimate the presence and severity of NAFLD, let alone they are validated in T1D populations (Marchesini et al., 2016). Indeed, ultrasonography only can reliably ascertain a fat infiltration of more than 20–30% of hepatocytes, while MRI-based techniques, such as proton density fat fraction, are excellent to determine and quantify even low-grade hepatic steatosis, but they are expensive, not widely available and cannot determine the concurrent presence of NASH or fibrosis (Marchesini et al., 2016; Gu et al., 2019). Keeping this difference in discriminating ability in mind, one would expect prevalence rates to be higher in the MRI studies, due to the limited detection capacity of ultrasound for low-grade steatosis. However, as seen in the meta-analysis of de Vries et al., pooled prevalence rates of MRI studies in subjects with T1D were lower, compared to those of ulftrasound-based studies (8.6 vs 27.1%) (de Vries et al., 2020). It is possible that referral bias partially explains this discrepancy since most ultrasound studies had retrospective designs, where only patients with T1D and liver abnormalities underwent abdominal ultrasound. Studies were also conducted primarily in tertiary centers. A third potential factor is that ultrasonographic studies may falsely classify patients as having NAFLD, when they actually might have had GlyH, mimicking steatosis on ultrasound.

It is indeed important to highlight the lack of prospective studies and the intrinsic risk of referral/selection bias in the ultrasound and biopsy-based studies since most studies originate from tertiary care centers and rely on retrospective analysis of those patients that had either imaging or biopsy. To strengthen this hypothesis, a very recent cross-sectional study, not included in the aforementioned meta-analysis, recruited 103 participants without any selection criteria besides T1D, excessive ethyl consumption, or the absence of secondary liver diseases to evaluate NAFLD prevalence (Barros et al., 2021). This study reported that the prevalence of steatosis was 12.6% based on classical ultrasound and 16.8% based on controlled attenuation parameter (CAP), a novel ultrasound-based marker of steatosis available on the Fibroscan^©^ device (Echosens, Paris, France). One important remark towards this study, in which NAFLD was only associated with the presence of the metabolic syndrome, is that the median hemoglobin A1c (HbA1c), a marker of longitudinal metabolic control, was 8.6% (IQR 2.1), which implies general poor metabolic control in this cohort. Another recently published study, investigated the association between visceral fat and NAFLD, assessed with MRI, in T1D. This was also a prospective study, without selection bias, that reported a rather low prevalence of NAFLD (11.6%) and an important association with visceral fat volume, stressing the association between metabolically unhealthy fat and NAFLD in T1D. Larger and unbiased population-wide studies are needed to address the epidemiology of NAFLD in T1D more accurately.

Currently, clinical awareness and systematic screening for NAFLD in T1D is globally virtually absent since knowledge of the impact of NAFLD on clinical outcomes in T1D is scarce. Unfortunately, NAFLD encompasses both a hepatic as a systemic health burden (Estes et al., 2018). Within the disease spectrum of NAFLD, liver steatosis can progress towards NASH, fibrosis, cirrhosis, and hepatocellular carcinoma (HCC), but it also poses an increased risk of cardiorenal morbidity and mortality (Marchesini et al., 2016). Epidemiological data on the progression rate towards end-stage liver disease are currently lacking in T1D, but evidence shows that in patients with NAFLD and T2D the risk of disease progression and overall and liver-related mortality is higher than in NAFLD patients without T2D (Stepanova et al., 2013). One study determined the natural history of patients with T1D and histologically proven chronic liver disease and found that 7% of patients with NAFLD developed cirrhosis over time. Furthermore, compared to the general population, there was a trend towards a 1.875-fold increased cirrhosis incidence in relatively young (<55 years) T1D patients, compared to the general population (Harman et al., 2014). Data on HCC in T1D-related NAFLD are absent to the best of our knowledge. Some studies have demonstrated associations between the presence of NAFLD and worse cardiorenal outcomes in T1D patients, but these studies originate from one research group only, meriting further confirmation in other cohorts (Targher et al., 2010; Targher et al., 2012a; Targher et al., 2012b; Mantovani et al., 2016). Nevertheless, these limited set of data indicate a potential association with hepatic and cardiometabolic risks, similar to findings in T2D cohorts, which might contribute to the relative excess mortality still present in T1D cohorts (Kasper et al., 2021; Przybyszewski et al., 2021). Therefore, NAFLD must be seen as an important potential hepatic and systemic complication in T1D patients.

### Fatty Liver Disease in Type 1 Diabetes: Pathophysiology

Several critical pathways are leading to diabetes-induced liver damage. Firstly, it is important to stress the differences, similarities, and overlaps between T1D and T2D, since both conditions are hallmarked by hyperglycemia, but have utterly different underlying mechanisms. T1D is, as mentioned above, a condition characterized by absolute insulin deficiency, while T2D primarily features insulin resistance, but can result in insulin deficiency as well when insulin production is depleted due to exhaustion. This means that most patients with T2D, in the early stages, are characterized by hyperinsulinemia, while insulin-dependent subjects with T2D and T1D are exposed to variable insulin gradients due to exogenous administration of insulin. Secondly, we emphasize the independent role of obesity and obesity-driven mechanisms of tissue damage, mostly, but not exclusively through means of insulin resistance.

Insulin resistance and hyperinsulinemia are the predominant causative factor for the development and progression of fatty liver disease (Marchesini et al., 1999; Buzzetti et al., 2016; Haas et al., 2016; Petta et al., 2016; Kitade et al., 2017; Manco, 2017; Gastaldelli and Cusi, 2019). While classically only causatively associated with T2D, recent studies have clearly indicated that visceral obesity, metabolic syndrome, and insulin resistance are increasingly common in T1D, so this mechanism is likely to occur in T1D as well (DeFronzo et al., 1982; Greenbaum, 2002; De Block et al., 2005; Stadler et al., 2010; Regnell and Lernmark, 2011; Donga et al., 2015; De Block et al., 2018; Priya and Kalra, 2018; American Diabetes Association, 2021a). Studies prospectively following new-onset patients with T1D, stratified by the presence of the metabolic syndrome at start or appearance thereafter, are therefore essential to disentangle the independent role of the metabolic syndrome on the development or course of metabolic complications, including NAFLD. It is important to mention that NAFLD is not always associated with the metabolic syndrome. A growing number of genetic risk alleles, including but not limited to PNPLA3 and TM6SF2, are associated with fatty liver disease as well (Eslam and George, 2020). Furthermore, these genetic contributions to NAFLD seem to exert a protective effect on cardiovascular risk, which is progressively associated with metabolic NAFLD (Stefan et al., 2019). Data on NAFLD risk alleles in T1D-associated NAFLD are lacking but are needed to fully appreciate the role of NAFLD in the cardiometabolic risk profile of patients with T1D.

In states of insulin resistance and hyperinsulinemia, liver lipogenesis is increased, while fatty acid oxidation and triglyceride secretion via very low-density lipoprotein (VLDL) are decreased. Dyslipidemia is further enhanced due to peripheral lipolysis increasing the circulating of free fatty acids (FFA) that are then taken in by the liver (Donnelly et al., 2005). Obesity, resulting in expansion of the visceral adipose tissue, especially when its maximum storage capacity is reached, results in lipotoxicity leading to even higher levels of FFA, ectopic fat accumulation, decreased levels of anti-inflammatory adipocytokines such as adiponectin, and higher levels of pro-inflammatory adipocytokines and chemokines leading to a chronic inflammatory state, further fueling insulin resistance (Van Gaal et al., 2006; Shoelson et al., 2007; Marseglia et al., 2014; Tarantino et al., 2019; Delli Bovi et al., 2021). Adiponectin is pivotal for hepatic insulin sensitivity and is lower in patients with NAFLD compared to those without (Francque et al., 2012; Shabalala et al., 2020). Obesity and overnutrition also lead to increased oxidative stress, reflecting the imbalance between production and removal of reactive oxygen species (ROS), which plays a key role in the development of insulin resistance and liver tissue damage (Marseglia et al., 2014; Delli Bovi et al., 2021). Furthermore, hepatic lipid overload induces overproduction of ROS, leading to intracellular damage and dysfunction affecting amongst others insulin signaling (Rolo et al., 2012; Tarantino et al., 2019). Nevertheless, antioxidant treatment of obesity or NAFLD thus far has shown little success (Sanyal et al., 2010; Rolo et al., 2012) and the antioxidant vitamin E is only recommended for nondiabetic patients with NAFLD.

The immune system is also involved, with macrophages/Kupffer cells, natural killer cells, and T-cells adding to the pro-inflammatory state and the development of NASH (Kitade et al., 2017; Van Herck et al., 2019; Vonghia et al., 2019). Immune activation will also further increase systemic insulin resistance creating a vicious cycle (Chen et al., 2017). Additionally, the liver is susceptible to hyperglycemia-induced oxidative stress involving the diacylglycerol (DAG)–protein kinase C (PKC)–NADPH-oxidase axis, leading to liver injury (Lucchesi et al., 2013; Gargouri et al., 2016; Volpe et al., 2018).

Another factor more specifically for insulin-dependent diabetes is the altered dynamic of insulin delivery and clearance in case of exogenous insulin administration. Blood from the portal vein mixes with blood from the hepatic artery in the liver sinusoids where, due to endothelium fenestrations, insulin can move freely to the space of Disse. Once it reached the space of Disse, insulin can be absorbed by the hepatocytes. Upon its secretion from the pancreas into the portal circulation, approximately 50–80% of insulin is cleared during first-pass transit through the liver, primarily by a receptor-mediated process carried out by hepatocytes (Polonsky et al., 1988; Najjar and Perdomo, 2019). Smaller amounts are degraded by Kupffer cells (15%), while non-receptor-mediated pinocytosis of insulin by hepatocytes may significantly increase insulin uptake, hence first pass elimination, at high circulating insulin concentrations (Duckworth et al., 1998). Intracellularly, the insulin-degrading enzyme (IDE) plays a major role in actual insulin clearance. Models have shown that patients with T2D have lower IDE levels contributing to hyperinsulinemia (Valera Mora et al., 2003; Pivovarova et al., 2015; Merino et al., 2020).

Insulin release from β-cells in healthy individuals is biphasic: following an increase in glucose, there is an initial peak secretion followed by a second excretion with slower progression to maximal secretion levels, persisting until glucose is cleared to normal (Rorsman et al., 2000). Circulating insulin levels are known to oscillate with high frequency. This is important since continuous insulin exposure downregulates insulin receptor density of target cells. The less variation in oscillation, the more insulin is needed to provide cellular effects (Bergsten, 2000). Subcutaneously administered insulin is absorbed in the bloodstream, hampers this oscillation, and only a fraction of it reaches the liver via the portal vein compared to normal conditions (Regnell and Lernmark, 2011). This altered kinetic implies the development of a relative insulin resistance and an increased insulin need, accompanied by systemic and hepatic effects. Diabetic rat models have shown that in the liver expression of GLUT2, a glucose transporter, is increased in states of hyperglycemia, but can be corrected to normal when euglycemic conditions are reached (Burcelin et al., 1992). However, due to potential upregulation of GLUT2, especially within patients with poor metabolic control, glucose gets transported into the liver, where it is converted into fat, contributing to hepatic steatosis. If fat accumulation in T1D, and also in insulin-dependent T2D, is indeed mediated by conversion from carbohydrates, aberrations in glycogen metabolism are also likely to be present in the liver. Indeed, insulin downregulates hepatic gluconeogenesis in normal conditions by downregulation of phosphoenolpyruvate carboxykinase and glucose-6-phosphatase and stimulates glycogen synthetase activation leading to glycogen synthesis (Han et al., 2016; Zhang et al., 2018a). When glycogen synthesis pathways are saturated due to long-lasting hyperglycemia, excess glucose is shunted away to lipogenic pathways (Flatt, 1995).

A very important question to address is the intrahepatic paradox of insulin resistance. Since insulin normally suppresses gluconeogenesis and promotes *de novo* lipogenesis, insulin resistance at the level of the liver would normally lead to both ineffective hampering of gluconeogenesis in combination with inhibition of lipogenesis (Brown and Goldstein, 2008; King et al., 2016). Thus, selective insulin resistance in fatty liver disease causes continuous hepatic glucose production, while the insulin sensitivity for lipid pathways leading to lipogenesis paradoxically remains intact (Santoleri and Titchenell, 2019). A recent study tested this hypothesis in obese humans with NAFLD and found that acute increases in lipogenesis are not explained by altered molecular regulation of lipogenesis through a paradoxical increase in lipogenic insulin action (Ter Horst et al., 2021). They suggest that increases in lipogenic substrate availability may be the key. Whether this applies as well in individuals with T1D is unknown.

Insulin influences intrahepatic fat synthesis by increasing sterol regulatory element-binding proteins (SREBPs) in hepatocytes (Dif et al., 2006) and their activity via upregulation of upstream stimulators of SREBP (Sun et al., 2016; Steensels et al., 2020). SREBPs are transcription factors activating the expression of various genes involved in the synthesis and uptake of cholesterol, fatty acids, triglycerides, and phospholipids (DeBose-Boyd and Ye, 2018). The SREBP-1c protein is essential for glucokinase, liver-type pyruvate kinase (LPK), fatty acid synthase (FAS), and acetyl-CoA-carboxylase (ACC) expression, and is upregulated in the presence of hyperglycemia (Shimomura et al., 1999; Bertolio et al., 2019). While LPK is mostly involved in converting phosphoenolpyruvate to pyruvate, the primary source for acetyl-CoA used for fatty acid synthesis, FAS and ACC exhibit effects on fatty acid transport to mitochondria, reducing mitochondrial fatty acid oxidation (Koo, 2013). An additional transcription factor, namely carbohydrate response element-binding protein (ChREBP), can also stimulate LPK gene transcription. Upregulation of this reaction happens also in hyperglycemic states but is not dependent on insulin (Iizuka and Horikawa, 2008). Therefore, both SREBPs and ChREBP are potential pivotal contributors to fatty liver disease in T1D and insulin-dependent T2D (Regnell and Lernmark, 2011; Benhamed et al., 2012).

Not only caloric excess but also the composition of nutrients is of importance. In Western diets, excessive consumption of fructose, as found in industrially processed foods and soft drinks, is often present. Fructose is considered partially responsible for fat accumulation and progression towards NASH (Brown et al., 1997; Lim et al., 2010; Jegatheesan and De Bandt, 2017). Chronic fructose consumption leads to activation of the abovementioned SREBP-1c and ChREBP provoking hepatic energy homeostasis (Roeb and Weiskirchen, 2021). Furthermore, fructose is broken down into pyruvate faster than glucose, leading to more substrate availability for *de novo* lipogenesis. Applied on patients with T1D, dietary restrictions are the first step in treatment, implying that diets rich in fructose are often avoided by these patients. However, hypoglycemia, which still occurs daily in the majority of individuals with T1D, demands swift treatment with sugar-rich beverages such as soft drinks and/or ingestion of industrially processed food, often rich in fructose. As a consequence, weight gain due to hypoglycemia-induced defensive snacking is an important factor contributing to overweight or obesity (Bumbu et al., 2018). Therefore, in individuals with T1D, especially when insulin therapy is not optimally tailored, weight gain due to energy- and fructose-rich defensive snacking could be a specific additional pathway leading to NAFLD susceptibility.

Besides the absence of insulin production and the effects of insulin replacement therapy, T1D features dysregulation of other pancreatic hormones that might contribute to NAFLD. Glucagon is a peptide hormone produced by α-cells in the pancreas, counteracting the effects of insulin to secure glucose homeostasis. Glucagon secretion is normally suppressed by hyperglycemia and the paracrine function of insulin, the latter not achieved with exogenous insulin, partially explaining hyperglucagonemia seen in T1D (Unger and Orci, 2010). Furthermore, amylin secretion is lost, which contributes to hyperglucagonemia. Amylin is a polypeptide hormone secreted by pancreatic β-cells in conjunction with insulin in response to nutrient stimuli (Edelman and Weyer, 2002). In normal conditions, amylin complements the former by suppressing glucagon secretion, resulting in the further suppression of hepatic glucose production in the presence of a postprandial glucose load, combined with slowing of gastric emptying to avoid postprandial glucose excursions (Nyholm et al., 1999; Edelman and Weyer, 2002; Fineman et al., 2002). T1D is therefore characterized by a paradoxical postprandial increase in glucagon, with on top of that a blunted glucagon response to hypoglycemia (Brown et al., 2008; Thivolet et al., 2019). Studies with pramlintide, a synthetic amylin analog, showed improvements in metabolic control, while a recent study with a dual hormone (insulin and pramlintide) artificial pancreas also showed improved time-in-range, indicating more time spent in optimal glycemic control (Singh-Franco et al., 2007; Haidar et al., 2020). Glucagon increases hepatic lipolysis and fatty acid oxidation, while it inhibits hepatic lipogenesis and the secretion of triglycerides and VLDLs (Galsgaard et al., 2019). Thus, glucagon could exert a protective effect on the liver. However, recent insights suggest the occurrence of hepatic glucagon resistance in patients with NAFLD, which promotes liver steatosis and hyperglycemia (Galsgaard, 2020). Evidence in T1D patients is lacking, but it can be hypothesized that in states of basal hyperglucagonemia, subsequent hepatic glucagon resistance will further contribute to worsening of both glycemic control and NAFLD. Glucagon and amylin therapeutics were tested in T1D models mostly for glycemic control, but further studies are needed to determine whether amylin could play a role in metabolic endpoints for patients with T1D since preliminary results showed weight loss in patients with T1D treated with pramlintide (Dunican et al., 2010).

Glucagon-like peptide 1 (GLP-1) is a hormone of the incretin system that is secreted upon food intake and acts on satiety, gastric emptying, and glycemia. GLP-1 levels are lower in T1D patients, while differences were also noted between C-peptide positive and negative individuals with T1D. The levels of GLP-1 correlate with glucagon values and the presence of the metabolic syndrome (Blaslov et al., 2015; Zibar et al., 2015; Thivolet et al., 2019). Due to their pleiotropic glucose-dependent effects that improve glycemic control and reduce body weight, GLP-1 analogs are currently approved in T2D, but not in T1D despite the presence of a deficiency and favorable results in trials with liraglutide (Dimitrios et al., 2020). In NAFLD cohorts, GLP-1 agonists reduce liver fat content and reduce NASH activity (Mantovani et al., 2021). Whether liver steatosis and GLP-1 levels are related in T1D-associated NAFLD is unexplored, but the potential add-on effects of GLP-1 agonists on liver fat content and NASH, on top of glycemic control and weight could aid in the debate of supportive GLP-1 therapy in patients with T1D, especially in those with metabolic syndrome.

It can be stated that insulin highly mediates intrahepatic fat homeostasis and can be linked to fatty liver disease. Fatty liver disease negatively affects insulin clearance and sensitivity, creating a vicious circle (Kotronen et al., 2007). The addition of insulin therapy in T2D subjects reduces liver fat (Juurinen et al., 2007). Pooled analysis showed that the HbA1c level was 2.7 mmol/mol lower in T1D subjects without NAFLD compared to those with NAFLD (de Vries et al., 2020). However, meta-regression analysis did not find an association between HbA1c and NAFLD (de Vries et al., 2020). This can hypothetically be explained based on the mechanisms outlined before since HbA1c only informs about mean blood glucose levels over a 2–3 month period, it cannot distinguish between patients with high variability in blood glucose compared to low differences in blood glucose, while upregulation of fatty-liver inducing molecules is dependent on not only times spent in high glycemia, but also fluctuations in glucose and insulin levels. The novel diabetes parameters time in range (TIR: 70–180 mg/dl) and coefficient of glycemic variation are continuous glucose monitoring-derived variables showing the time spent in ideal glucose range and the oscillation throughout the day, respectively. Further research is needed to confirm whether a lower TIR and higher glucose variability are associated with a higher hepatic fat content.

### From Non-Alcoholic Fatty Liver Disease to Metabolic Dysfunction-Associated Fatty Liver Disease: Possible Consequences of a Paradigm Shift

Recently, the term metabolic dysfunction-associated fatty liver disease (MAFLD) was coined as an alternative to NAFLD to better reflect the current knowledge on the factors driving NAFLD as well as to address existing issues with the definition of NAFLD, such as the exclusion of other chronic liver diseases while they actually might co-exist (e.g. chronic viral hepatitis and NAFLD) and subsequent consequences on studies and trials (Eslam et al., 2020; Ratziu et al., 2020). The MAFLD criteria dictate that when hepatic steatosis is present in adults, MAFLD is diagnosed when: 1) overweight or obesity is present or 2) T2D is present or 3) at least two risk abnormalities are present including elements of the metabolic syndrome, insulin resistance according to the homeostasis model (HOMA-IR) or C-reactive protein levels above 2 mg/L (Eslam et al., 2020).

Applying the MAFLD nomenclature and definitions is potentially not without problems in T1D. Firstly, the criteria imply that overweight patients with T1D automatically meet the MAFLD criteria when steatosis is present based on imaging techniques, biomarkers, or histology. As we will see below, this might lead to the erroneous diagnosis of MAFLD since ultrasound, the most used imaging tool, cannot distinguish reliably between steatosis and other hepatopathies (Zhang et al., 2018b). Additionally, the accuracy of NAFLD biomarkers is unexplored in T1D cohorts, but two studies found very high and incongruent prevalence rates based on multiple NAFLD scoring systems, indicating the possibility of overestimation and the need for cross-validation (Singh et al., 2018; Sviklāne et al., 2018). Secondly, in several lean subjects with T1D, some criteria of the metabolic syndrome, such as arterial hypertension and dyslipidemia, may be a consequence of diabetes itself (e.g. T1D-induced nephropathy) rather than reflecting underlying metabolic disease impeding proper use of these criteria (Chillarón et al., 2011; Vergès, 2020). On the other hand, the HOMA-IR model cannot be used in T1D because of absolute insulin deficiency, reducing the number of applicable risk factors for these patients. Lastly, patients with co-existent liver disease or excessive alcohol intake are excluded according to NAFLD criteria but are included according to MAFLD criteria. Therefore, studies are needed comparing NAFLD and MALFD criteria in T1D cohorts to evaluate the clinical impact of both definitions.

### Glycogenic Hepatopathy: Epidemiology

**FIGURE 1:**
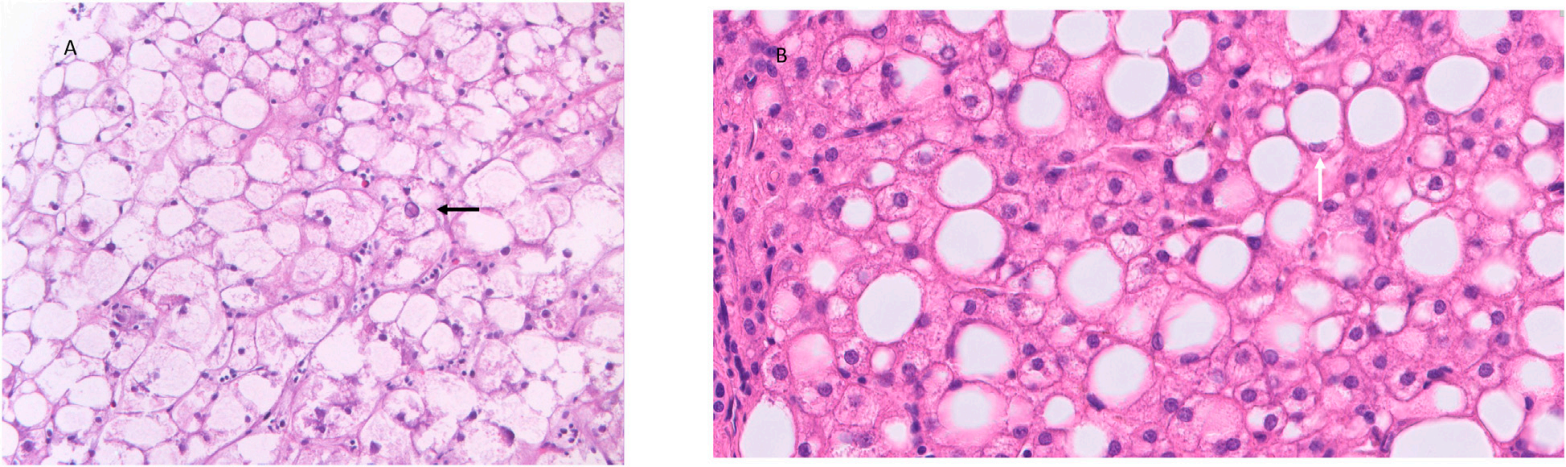
Histology features of GlyH and liver steatosis. Caption: **(A)** The liver parenchyma in GlyH is composed of enlarged, swollen hepatocytes with a pale cytoplasm due to the accumulation of glycogen. In the center a hepatocyte with a glycogenated nucleus can be observed (black arrow) (HE, 40x). **(B)** The liver parenchyma shows several steatotic hepatocytes, corresponding to macrovesicular steatosis (HE, 20x). A steatotic hepatocyte has a compressed nucleus, surrounded by an “empty” cytoplasm. The empty appearance of the cytoplasm is due to the dissolution of the fatty acids in the cytoplasm during the technical process (white arrow) (HE, 40x).

GlyH is a rare clinical condition that is hallmarked by excessive accumulation of glycogen in the hepatocytes. It is seen in patients, mostly children and adolescents, with poorly controlled T1D, and is mostly described in case reports and case series (Table 1). This hepatopathy was initially described as part of Mauriac syndrome, a rare complication of poorly controlled T1D, with extreme liver enlargement due to glycogen deposition, growth failure, cushingoid features and delayed puberty. The term GlyH was later coined to address liver disease in diabetes characterized by mere glycogen overload attributable to poor glycemic control, without other features of Mauriac syndrome (Torbenson et al., 2006). The large majority of cases of GlyH are seen in uncontrolled T1D, but rare reports in T2D patients, mostly when insulin therapy is present, are also made (Olsson et al., 1989; Tsujimoto et al., 2006; Umpaichitra, 2016; Kumar et al., 2018). The gold standard to diagnose GlyH is liver histology since no serologic test nor specific imaging study is currently available to accurately diagnose GlyH (Sherigar et al., 2018). Clinical and biochemical features, supported by imaging studies can however guide the physician towards GlyH, but awareness is needed since the manifestation of GlyH is very similar to NAFLD, and occurs in the same subtype of patients (see Table 2). A liver biopsy will typically show swollen hepatocytes with cytoplasmatic glycogen accumulation. Associated steatosis is often described, stressing the clinical overlay of both hepatopathies (Figure 1). A hematoxylin and eosin stain of the specimen shows pale, enlarged hepatocytes with prominent plasma membranes, increased cytoplasmic volume, and empty, glycogenated nuclei featuring ring-like chromatin elements (Sherigar et al., 2018). Adding diastase to the Periodic-Acid Schiff stained specimen, enabling digestion of glycogen in the hepatocytes, would lead to the appearance of “ghost cells” (Shah et al., 2017). It is important to mention, that in the histology of NAFLD, besides steatosis, inflammation, and fibrosis, hepatocellular glycogenated nuclei are also often described, facilitating differentiation between NASH and alcoholic steatohepatitis, since they are rarely seen in the latter (Pinto et al., 1996; Takahashi and Fukusato, 2014). Furthermore, the occurrence of glycogenated nuclei in NAFLD cases is associated with disease progression, which may be reflected in the number of cases of concomitant NASH in GlyH case reports (Table 1) (Schwertheim et al., 2020).

**TABLE 1:** Summary of published cases of GlyH in T1D.

Author	N	Age group	Clinical features			Metabolic control (HbA1c)^a^	Histologically confirmed GlyH?
			Hepatomegaly	Elevated transaminases	Hyperechogenicity		
Evans et al. (Evans et al., 1955)	4	Mixed	4/4	NA	NA	Poor (NA)	Yes
Ruschhaupt and Rennert (Ruschhaupt and Rennert, 1970)	1	Children	1/1	1/1	NA	NA	Yes
Berman (Berman, 1973)	1	Adults	1/1	1/1	NA	NA	Yes
Olsson et al. (Olsson et al., 1989)	4	Mixed	NA	4/4	0/2*	Poor (NA)	Yes
Chatila and West (Chatila and West, 1996)	11	Adults	9/11	9/11	NA	Poor (NA)	Yes
Munns et al. (Munns et al., 2000)	3	Adolescents	3/3	3/3	1/2*	Poor (14.1; 13.3,12.2%)	Yes
Torres and Lopez (Torres and López, 2001)	1	Adults	1/1	1/1	1/1	Poor (8.1%)	Yes
Carcione et al. (Carcione et al., 2003)	2	Children	2/2	2/2	NA	Poor (NA)	Yes
Torbenson et al. (Torbenson et al., 2006)	14	Mixed	9/14	12/14	NA	Poor in all (only few cases had a mentioned HbA1c, ranging from 9.9 to 13.5%)	Yes ^a^ steatosis present in 2 cases, NASH in 1
Sayuk et al. (Sayuk et al., 2007)	2	Adolescents	2/2	2/2	2/2	Poor (8.1%,16.0%)	Yes
Cuthbertson et al. (Cuthbertson et al., 2007)	1	Adolescents	1/1	1/1	1/1	Poor (12.2%)	Yes
Basset et al. (Bassett et al., 2008)	1	Children	1/1	1/1	0/1	Poor (10.1%)	Yes
Martocchia et al. (Martocchia et al., 2008)	1	Children	1/1	1/1	1/1	Poor	Yes ^a^ steatosis
Hudacko et al. (Hudacko et al., 2008)	1	Adolescents	1/1	1/1	1/1	Poor (13.3%)	Yes
Abaci et al. (Abaci et al., 2008)	1	Adolescents	1/1	1/1	1/1	Poor (11.1%)	Yes ^a^ steatosis
Van den Brand et al. (van den Brand et al., 2009)	1	Adults	1/1	1/1	NA	Poor (15.3%)	Yes
Sweetser and Kraichely (Sweetser and Kraichely, 2010)	1	Adults	1/1	1/1	NA	Poor (15.0%)	Yes
Saxena et al. (Saxena et al., 2010)	1	Adults	1/1	1/1	1/1	Poor (13.7%)	Yes
Bua et al. (Bua et al., 2010)	1	Adolescents	1/1	1/1	0/1	Poor (12.0%)	Yes
El Karaksy et al. (El-Karaksy et al., 2010)	60/692	Children	13/60	27/60	17/60	Poor compared to controls	5 biopsies performed, 3 in patients with signs of GlyH: 3/3 had GlyH, 1/3 had steatosis
Aljabri et al. (Aljabri et al., 2011)	1	Children	1/1	1/1	1/1	Poor (13.0%)	Yes
Murata et al. (Murata et al., 2012)	1	Adults	1/1	1/1	1/1	New-onset diabetes 6.2%)	Yes
Dantuluri et al. (Dantuluri et al., 2012)	1	Children	1/1	1/1	NA	Poor (11.0%)	Yes
Saadi (Saadi, 2012)	1	Adolescents	1/1	1/1	1/1	Poor (11.0%)	Yes
Lin et al. (Lin and Kao, 2012)	1	Children	1/1	11	NA	Poor (12.8%)	Yes
Messeri et al. (Messeri et al., 2012)	1	Adults	1/1	1/1	1/1	Poor (10.3%)	Yes ^a^ steatosis
Al-Hussaini et al. (Al-Hussaini et al., 2012)	22/106	Children	10/22	0/22	12/22	Case group had worse HbA1c: 12.14 vs 10.7%	No biopsies performed
Imtiaz et al. (Imtiaz et al., 2013)	1	Adolescents	1/1	1/1	NA	Poor (14.6%)	Yes
Saikusa et al. (Saikusa et al., 2013)	2	Children	2/2	2/2	NA	Poor (12.0%)	No biopsies were taken, differentiation with NAFLD was done with dual-echo MRI.
Cha et al. (Cha et al., 2013)	3	Adults	1/1*	3/3	3/3	Poor (13.8, 12.9, 13.6%)	Yes
Butts et al. (Butts et al., 2014)	1	Children	1/1	1/1	1/1	Poor (8.8%)	Yes ^a^ steatosis
Fitzpatrick et al. (Fitzpatrick et al., 2014)	31	Children	31/31	31/31	26/31	Poor (mean: 11.0%)	19 biopsies performed. GlyH was present in 17 cases, inflammation in 8, mild fibrosis in 14
Jeong et al. (Jeong et al., 2014)	1	Children	1/1	1/1	NA	Poor (10.7%)	Yes
Martin and Tomlinson (Martin and Tomlinson, 2014)	1	Children	1/1	1/1	NA	Poor (10.4%)	Yes
Parmar et al. (Parmar et al., 2015)	1	Adults	1/1	1/1	NA	Poor (NA)	Yes
Atmaca et al. (Atmaca et al., 2015)	1	Adolescents	1/1	1/1	1/1	Poor (NA)	Yes
Brouwers et al. (Brouwers et al., 2015)	4	Adolescents	4/4	4/4	NA	Poor (9.5%)	Yes
Garcia-Suarez et al. (García-Suárez et al., 2015)	1	Adults	1/1	1/1	1/1	Poor (10.5)	Yes
Irani et al. (Irani et al., 2015)	1	Adolescents	1/1	1/1	1/1	Poor (12.0%)	Yes
Xu et al. (Xu et al., 2015)	1	Adults	1/1	1/1	NA	Poor (14.6%)	Yes
Deemer and Alvarez (Deemer and Alvarez, 2016)	1	Adolescents	1/1	1/1	1/1	Poor (11.3%)	Yes
Silva et al. (Silva et al., 2016)	4	Adults	4/4	4/4	3/4	Poor (9.0, 10.1, 10.9, 15.7%)	Yes
Chandel et al. (Chandel et al., 2017)	1	Children	1/1	1/1	NA	Poor (10.5%)	Yes
Ikarashi et al. (Ikarashi et al., 2017)	4	Adults	4/4	4/4	NA	Poor (11.7, 11.0, 13.6, 16.5%)	Yes ^a^ NASH in 3/4 cases
Mukewar et al. (Mukewar et al., 2017)	36	Mixed	22/36	NA	NA	Poor (mean: 11.4%)	20 biopsies were performed, all positive for GlyH
Al Sarkhy et al. (Al Sarkhy et al., 2017)	1	Children	1/1	1/1	0/1	Poor (11.6%)	Yes
Shah et al. (Shah et al., 2017)	1	Adults	1/1	1/1	NA	NA	Yes
Maharaj et al. (Maharaj et al., 2017)	1	Adults	1/1	1/1	NA	Poor (12.3%)	Yes ^a^ signs of drug-induced liver injury
Asada et al. (Asada et al., 2018)	1	Adults	1/1	1/1	1/1	Poor (12.9%)	Yes ^a^ NASH
Abboud et al. (Abboud et al., 2018)	1	Adolescents	1/1	1/1	0/1	Poor (12.4%)	Yes ^a^ NASH
Glushko et al. (Glushko et al., 2018)	1	Adolescents	1/1	1/1	1/1	Poor (14.6%)	Yes
Lombardo et al. (Lombardo et al., 2019)	1	Children	1/1	1/1	0/1	Poor (13.1%)	Yes (as part of Mauriac syndrome)
Patita et al. (Patita et al., 2019)	1	Adults	1/1	1/1	0/1	Poor (14.8%)	Yes ^a^ steatosis
Aydin et al. (Aydın et al., 2019)	17/110	Children and adolescents	NA	Not significantly higher compared to controls	17/17	Mean HbA1c 10.1% in cases, 11.8% in controls	No biopsies were described
Azariadis et al. (Azariadis et al., 2019)	1	Adolescents	1/1	1/1	0/1	Poor (11.2%)	Yes
Lui et al. (Lui et al., 2019)	1	Adults	1/1	1/1	0/1	Poor (18.7%)	Yes
Medhioub et al. (Medhioub et al., 2019)	1	Adolescents	1/1	1/1	01	Poor (10.5%)	Yes
Sharma et al. (Sharma et al., 2019)	1	Adolescents	1/1	1/1	NA	Poor (12.1%)	Yes
Regan et al. (Regan et al., 2020)	1	Adults	NA	1/1	NA	Poor (13.0%)	Yes
Aluko et al. (Aluko et al., 2020)	1	Adults	1/1	1/1	NA	Poor (11.5%)	Yes
Alenazy et al. (Alenazy et al., 2020)	1	Adolescents	1/1	1/1	1/1	Poor (11.5%)	Yes
Fujisaki et al. (Fujisaki et al., 2020)	1	Adult	1/1	1/1	1/1	No HbA1c provided, poor control mentioned in text	Yes
Hsu et al. (Hsu et al., 2021)	1	Adults	NA	1/1	0/1	NA	Yes
Abu et al. (Abu et al., 2021)	2	Children	2/2	2/2	0/2	Poor (case 1 ranging from 12.5 to 16.1%, case 2 ranging from 10.0 to 13.3%)	Yes
Adams et al. (Adams et al., 2021)	1	Adolescents	1/1	1/1	1/1	Poor (13.5)	Yes ^a^ steatosis
Azhar et al. (Azhar et al., 2021)	1	Adults	1/1	1/1	1/1	Poor (9.5–11%)	Yes
Ahmed et al. (Ahmed et al., 2021)	1	Adolescents	1/1	1/1	1/1	Poor (14.0%)	Yes ^a^ steatosis and fibrosis
Fox et al. (Fox et al., 2021)	1	Children	1/1	1/1	1/1	Poor (14.0%)	Yes (as part of Mauriac syndrome)

a Reflected by a HbA1c > 8%; *: ultrasound is mentioned, but echogenicity of the liver is unmentioned, and therefore probably normal; GlyH: glycogenic hepatopathy.

**TABLE 2:** Clinical differentiation between NAFLD and GlyH in T1D.

	NAFLD	GlyH
Age group	No specific age group	Both children, adolescents and adults can be affected, although most reported cases are in adolescents due to intrinsic difficulties of obtaining metabolic control in this subgroup
Gender	No known sex differences	No known sex differences
Anthropometric features	Metabolic syndrome often present, often abdominal adiposity. Metabolic control is often poor, but not so distinctively deteriorated as in GlyH	There is a strong association with (often extremely) poor metabolic control, but not with the presence of the metabolic syndrome nor abdominal obesity
Abdominal discomfort	Can be present due to liver capsule distention, but is rather uncommon	Often described due to rapid liver capsule distention
		Nausea and vomiting is described, often in cases were diabetic ketoacidosis is present
Physical examination	Signs of hepatomegaly may be present. Signs of liver cirrhosis might be present in advanced NAFLD.	Tender hepatomegaly is described
Ascites	May be present in NAFLD-cirrhosis. No reports of ascites in T1D patients available	Rarely seen, due to sinusoidal compression by swollen hepatocytes due to glycogen accumulation. No doppler studies available to evaluate the presence of ultrasonographic features of sinusoidal obstruction
Liver enzymes	Normal or mildly elevated	Mild to moderate/severe elevation of ALT, AST, with predominant elevation in AST levels, although a mixed or predominantly cholestatic pattern can occur. Marked elevations in the range of 100-fold the upper limit of normal are reported
Lactate levels	Normal	Elevation is described, often in patients with ketoacidosis, but also in those without
Liver synthetic function	Preserved	Preserved
Ultrasonography	Hyperechogenic compared to the right kidney parenchyma	Hyperechogenic compared to the right kidney parenchyma. Hepatomegaly is commonly present
	Severe steatosis features echo-beam attenuation with loss of diaphragm visibility and loss of peripheral portal vein visibility	
	Hepatomegaly is possible due to fatty infiltration, but usually less pronounced compared to GlyH	
CT	Hypodense compared to the spleen, indicative of steatosis	Hyperdense compared to the spleen, although co-existent steatosis can attenuate this contrast effect. One case mentioned multiple arterial-enhancing hepatic nodules (Hsu et al., 2021)
MRI	Difference in the intensities of T1 weighted gradient-dual-echo MRI images with in-phase and opposed-phase conditions indicative for steatosis	No difference in the intensities of T1 weighted gradient-dual-echo MRI images with in-phase and opposed-phase conditions indicative for GlyH
Biopsy	Micro- or macrovesicular steatosis accompanied by inflammation, ballooning and/or fibrosis in NASH.	Hematoxylin and eosin (HE) stain would show pale and enlarged hepatocytes with prominent plasma membranes, increased cytoplasmic volume, and numerous glycogenated nuclei, which are empty nuclei with ring-like chromatin elements
	Rarely a glycogenated nuclei can be observed, which is potentially associated with progressive disease	Addition of diastase to the Periodic-Acid Schiff stained specimen causes enzymatic breakdown of glycogen in the hepatocytes

**FIGURE 2:**
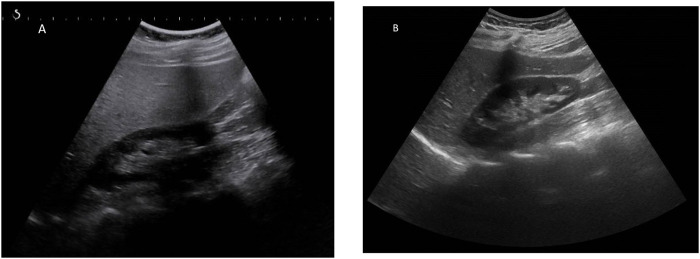
Ultrasonographic features seen in both GlyH and liver steatosis. Caption: **(A)** A greyscale ultrasound image of the liver parenchyma compared to the kidney will show hyperechogenicity of the liver in both GlyH and fatty liver disease, as seen in this 40-year old patient with T1D and NAFLD from our clinic. **(B)** In a normal liver, the echogenicity of liver and kidney parenchyma is similar, as seen in this 42- year old patient with T1D from our clinic.

**FIGURE 3:**
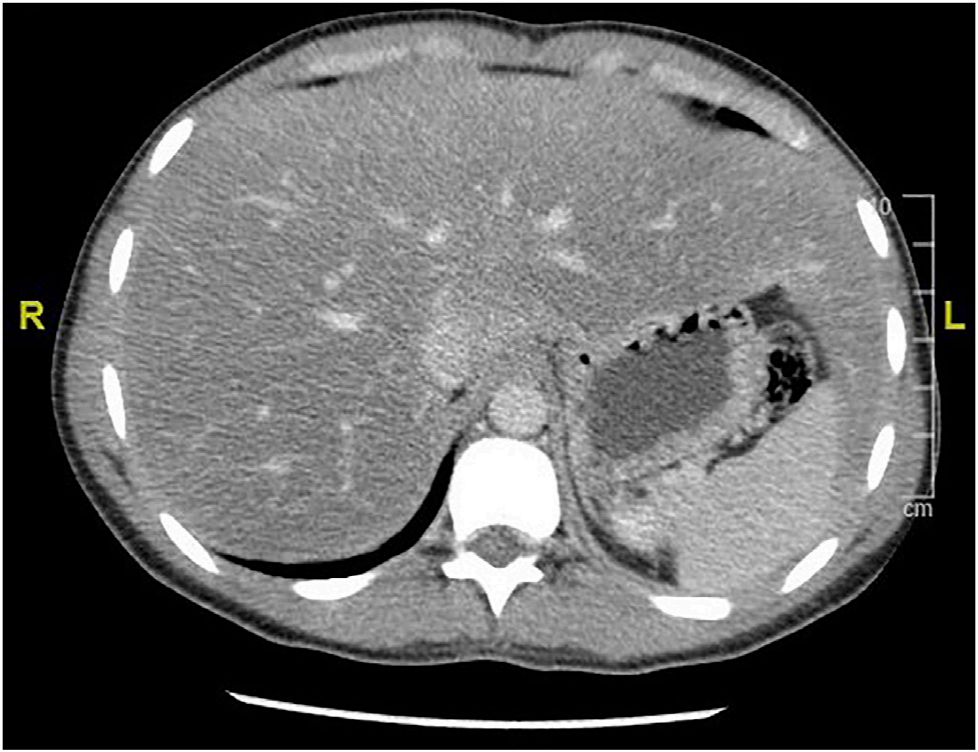
CT imaging features of liver steatosis. Caption: Transverse CT image of the liver showing decreased density of the liver compared to the spleen in this 38-year old patient with NAFLD. In GlyH, the inverse image can be witnessed with increased density compared to the spleen, but due to concomitant steatosis, this contrast is potentially attenuated in metabolic patients.

**FIGURE 4:**
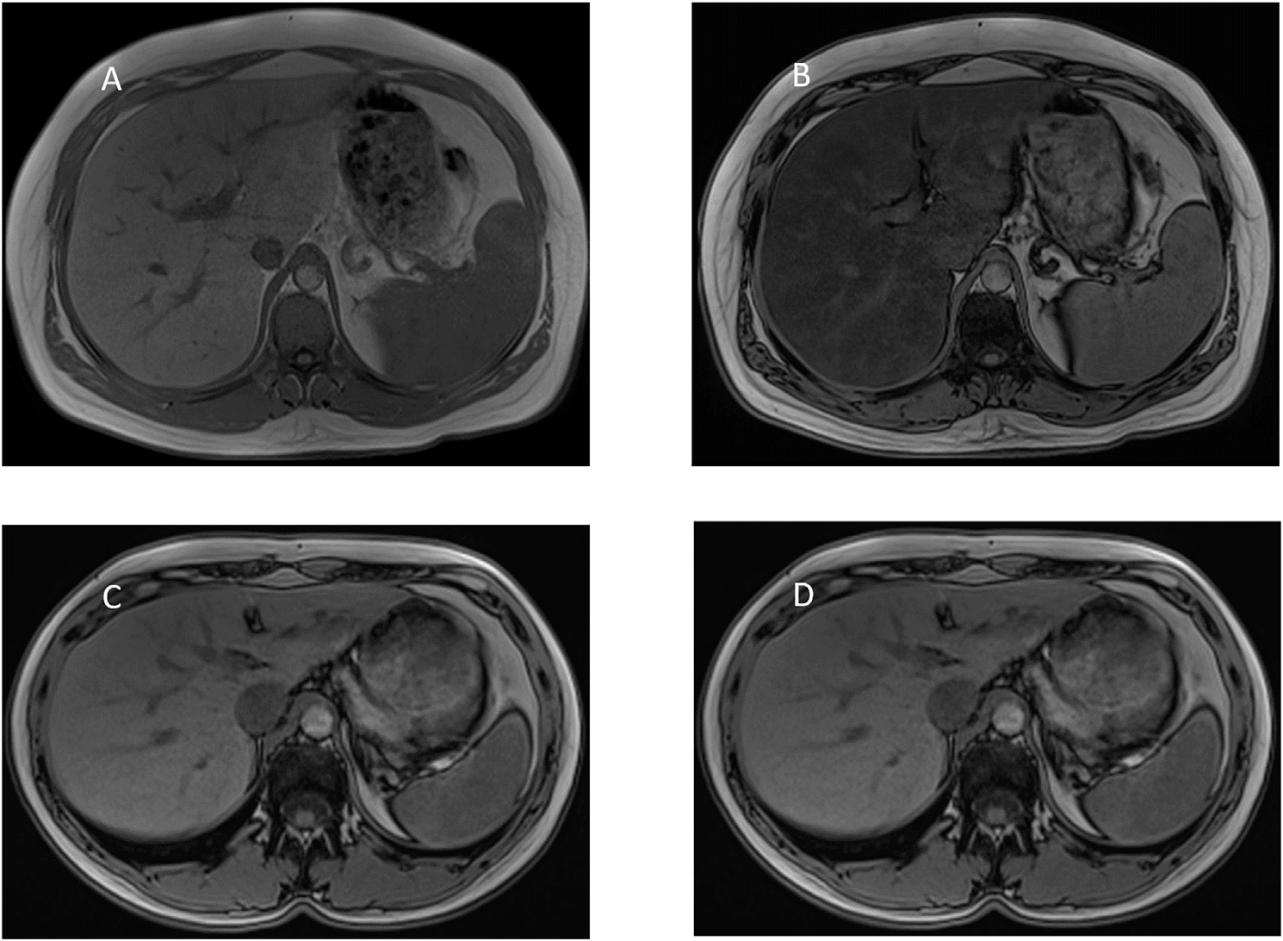
MRI-imaging features of liver steatosis. Caption: MRI proton chemical shift imaging with **(A)** in-phase T1-weighted gradient-echo image showing normal signal intensity of the hepatic parenchyma and **(B)** opposed-phase T1-weighted gradient-echo image showing a marked drop in signal intensity of the hepatic parenchyma, indicating diffuse hepatic steatosis. In contrast, MRI proton chemical shift imaging with **(C)** in-phase and **(D)** opposed-phase T1-weighted gradient-echo image showing normal signal intensity of the hepatic parenchyma indicating the absence of steatosis. In GlyH, as described by Saikusa et al., there is no drop in intensity either between in- and out-of-phase images.

GlyH and hepatic steatosis showcase important similarities on ultrasound imaging studies. Ultrasound studies will reveal hyperechogenic liver parenchyma and hepatomegaly, mimicking NAFLD (Figure 2). Moreover, criteria to define echogenicity or liver size are subjective and often ill-defined. Computed tomography (CT) could aid to differentiate between GlyH and NAFLD, since GlyH features an increased liver density on CT, whereas liver density is decreased in patients with NAFLD, especially compared to the density of the spleen (Figure 3) (Sweetser and Kraichely, 2010). However, CT diagnosis of NAFLD is not recommended in guidelines, nor studied in T1D cohorts, so the utility of CT needs further validation, especially due to the radiation burden associated with CT, which is not present in MRI-based imaging (see Table 2) (Marchesini et al., 2016; Chalasani et al., 2018; de Vries et al., 2020). Furthermore, as mentioned above, and in Table 1, steatosis is often co-present in GlyH and vice versa, potentially attenuating the contrast on CT and impeding diagnosis. Neither CT nor ultrasound are thus useful tests for the definitive diagnosis of GlyH. However, MRI-based imaging studies might be beneficial, since MRI can distinguish fat from glycogen deposition or acute tissue injury. MRI imaging in GlyH shows low intensities on T2 weighted images, whereas T1 weighted gradient-dual-echo MRI images with in-phase and opposed-phase conditions could efficiently differentiate hepatic glycogen from liver steatosis (Table 2 and Figure 4) (Sweetser and Kraichely, 2010; Saikusa et al., 2013).

It is important to mention that almost all literature concerning NAFLD in T1D patients comes from ultrasound-based studies alone, while the majority of GlyH reports are based on initial ultrasound, followed by histology. One pediatric cross-sectional study addressed this issue by using well-defined ultrasound parameters in a cohort of 106 children with T1D. Twenty-one percent of cases had abnormal liver ultrasounds (hepatomegaly or hyperechogenicity) without underlying secondary causes of hepatopathy (Al-Hussaini et al., 2012). Those with hyperechogenicity had poorer glycemic control (mean HbA1c 12.14 vs 10.7%, *p*-value = 0.09), which is in line with the hypothesis that fat and/or glycogen depositions are likely to be causing the sonographic features, since their association with glycemic control in T1D subjects. It should also be noted, regarding the latter study, that both case and control groups display very poor glycemic control, which is not in line with current treatment standards (Lavens et al., 2021).

Whereas transaminase levels are often elevated in cases of GlyH, NAFLD patients do not always display an elevation of liver enzymes. One study compared T1D patients with and without hepatopathy (based on imaging, not on histology) and did not find significant correlations between ALT and AST levels and aberrant ultrasonographic imaging, but hyperechogenicity was indeed correlated to poor glycemic control (Aydın et al., 2019). One pooled analysis of 192 documented cases of histologically proven GlyH showed that both ALT and AST are moderate-to-severely elevated in 78 and 76% of cases, with an AST-predominant pattern and improvement when glycemic control was obtained (Haffar et al., 2021). In this pooled analysis, the median HbA1c was 12% and median BMI 21 kg/m^2^, stressing the correlation with extremely poor glycemic control, which is hardly seen any longer in current practice, but not necessarily with abdominal adiposity. To our knowledge, no published comparisons of transaminase levels are made between histology-proven NAFLD versus GlyH patients. The problem, therefore, remains that currently, liver biopsy is the only reliable way to differentiate between NAFLD or GlyH. However, it is known that GlyH is associated with extremely poor metabolic control, and is reversible when glycemia ameliorates, making it self-limiting. Therefore, physicians prefer to try to ameliorate the metabolic control and wait for the clinical image to recover, instead of performing liver biopsy. This makes the exact prevalence of GlyH extremely difficult to assess. Furthermore, especially in Western countries, due to the introduction of several technologies such as intermittent or continuous glucose monitoring devices and insulin pumps, the majority of T1D patients will not reach such dramatic glycemic control leading into GlyH. Finally, the ethical limitations of liver biopsy pose the same challenge to determine the exact prevalence of NAFLD. To date, there are no large epidemiological studies or meta-analyses available assessing the prevalence and incidence of GlyH in T1D. We have summarized all relevant case series and studies focusing on GlyH in T1D in Table 1.

### Glycogenic Hepatopathy: Pathophysiology

As mentioned above, it can be hypothesized that fatty liver disease in T1D originates partly from conversion from excess carbohydrates into fat. Therefore, it can be anticipated that glycogen metabolism is affected too. Indeed, glycogen accumulation is described in T1D, especially in younger patients with poor glycemic control.

Hepatocytes take up glucose, independently of insulin, by the low-affinity, high-capacity glucose transporter GLUT2, which facilitates the entry of glucose in the presence of high concentrations of glucose in liver sinusoidal blood. Glucose is then rapidly phosphorylated to glucose-6-phosphate by the hepatic hexokinase isoform glucokinase. From glucose-6-phosphate, glycogen is produced by the enzyme glycogen synthase, via the precursor uridine diphosphate (UDP)-glucose. Glycogen synthase exists in an active dephosphorylated, and active phosphorylated form. The active dephosphorylated structure of glycogen synthase is produced by the action of a phosphatase enzyme, which is stimulated by elevated glucose and insulin levels. (Roden and Bernroider, 2003; Regnell and Lernmark, 2011; Han et al., 2016; Petersen et al., 2017; Sherigar et al., 2018). In T1D, insulin deficiency leads to low hepatic glucokinase levels resulting in a decrease in glucose-to-glycogen shunting. This, together with elevated glucagon levels, will stimulate glycogenolysis and gluconeogenesis as mentioned above. The balance between hepatic glucose production and uptake will tilt towards output, unless insulin is administered, which leads to a normalization of glucose uptake by the hepatocyte and a rise in glycogen content, which will persist even when blood glucose levels shift again. As a result, hepatocyte glycogen accumulation is promoted by high cytoplasmatic glucose concentration in the presence of insulin. Of note, the insulin dose is often injected in order to correct the overt hyperglycemia, but is often not sufficient to restore adequate glycemic control. Additionally, hepatic glycogenolysis is inhibited in hyperglycemic conditions (Petersen et al., 1998). As seen in Table 1, a large proportion of GlyH cases occur during childhood or adolescence. This may be due to compliance issues in the early phases of therapy, often characterized by insufficient or absent insulin doses, leading to hyperglycemia, leading to correction efforts shifting towards supraphysiological insulin doses (Carcione et al., 2003). Furthermore, α-cell disturbances are associated with T1D, making individuals more prone to hypoglycemia due to compromised glucagon secretion. This might contribute to difficulties in maintaining glycemic control and tailoring insulin therapy as well (Yosten, 2018). The pivotal factor in the pathogenesis of GlyH is therefore a combination of severe fluctuation in levels of glucose and administration of infra- and supraphysiological levels of insulin to try and control the glycemia. It is unknown why only a small subset of patients develops GlyH, since glycemic fluctuations are so common in T1D patients. It is possible that the introduction of long-acting insulin has dramatically decreased the incidence of GlyH, due to its stabilizing effects (Sherigar et al., 2018). Another hypothesis is genetic variation in the genes that code for glycogen synthase, glucose-6 phosphatase activity or glycogen phosphorylase kinase such as PHKG2, which is described in Mauriac syndrome (MacDonald et al., 2016).

### Clinical Practice Recommendation

Since GlyH presents itself mostly with elevated liver enzymes, abdominal discomfort or clinical signs in the presence of very poor glycemic control, most cases will be discovered and referred to the pediatrician/hepatologist (Sherigar et al., 2018). However, on the one hand, as seen above, raised ALT and AST is not necessarily present, and, on the other hand, elevated liver enzymes can have many causes. Therefore, several authors recommend systematic screening for GlyH by means of abdominal ultrasound, but general guidelines are lacking (Al-Hussaini et al., 2012; Aydın et al., 2019). At present, international guidelines do not recommend standardized screening, with ultrasound or risk scores, for NAFLD in subjects with T1D (Leoni et al., 2018). Furthermore, it is generally accepted, that screening for NAFLD solely based on elevation of liver enzymes is futile, since even NASH can feature normal liver enzymes (Leoni et al., 2018; Ma et al., 2020). To date, there are no serological tests available to screen for NAFLD or for GlyH, nor to distinguish them. Therefore, regular ultrasound (e.g. very 2–3 years, based on NAFLD screening in general (Marchesini et al., 2016; Berzigotti et al., 2021)) screening could aid in timely discovery of hepatopathy, since both NAFLD and GlyH have similar ultrasound features. The discovery of a bright, hyperechogenic liver on a standard abdominal ultrasound would justify further work-up to differentiate between the two conditions. GlyH is considered benign and reversible upon amelioration of glucose control, but there are no pooled data concerning long-term outcomes. NAFLD is increasingly studied in T1D, and links are progressively made with an increased risk of cardiovascular and renal disease (Targher et al., 2010; Targher et al., 2012a; Targher et al., 2012b; Targher et al., 2014; Mantovani et al., 2016). Furthermore, the hepatic burden of NAFLD in T1D, including advanced fibrosis and hepatocellular carcinoma is largely unexplored as mentioned above. As proposed by several authors, magnetic resonance imaging could have a place in the work-up of hepatopathy in T1D, since it can distinguish between GlyH and NAFLD, and simultaneously grade the amount of fat accumulation sensitively (Sweetser and Kraichely, 2010; Murata et al., 2012; Saikusa et al., 2013; Gu et al., 2019). The problem with MRI is its costs and its availability. A promising novel asset is the controlled attenuation parameter (CAP), which is a continuous steatosis index based on TE, available on the Fibroscan^©^ device (Echosens, Paris, France), with good overall sensitivity and specificity (Myers et al., 2012; de Lédinghen et al., 2016). Although a recent analysis showed that it cannot be used to grade steatosis accurately, theoretically, CAP might be able to distinguish GlyH and NAFLD in cases of bright liver on ultrasound, but this is not yet studied (Petroff et al., 2021). Further research is therefore needed to determine distinct characteristics of GlyH or NAFLD in T1D, ideally noninvasively, to distinguish them easily and to avoid extensive workup including liver biopsy and associated costs. The potential of systematic screening of T1D patients, or selected screening based on the presence of the metabolic syndrome or poor metabolic control, with a combination of ultrasound and TE must be prospectively evaluated in longitudinal research, whether it could be of value to detect early hepatopathy.

Pragmatically, when faced with a T1D patient with suspected hepatopathy, a combination of clinical signs and imaging studies (see Table 2) could aid the hepatologist or endocrinologist to further determine the underlying condition. Based on this information, the likelihood for one or the other can be determined. Patients that are referred with elevated liver transaminases, certainly when accompanied with poor metabolic control, should be screened for concomitant liver disease (viral hepatitis, autoimmune hepatitis, Wilson’s disease) using laboratory tests and both ultrasound and TE, to determine the possibility of GlyH and/or NAFLD and to look for other potential structural abnormalities. When hepatopathy is present, we would suggest to invest in intensive education and insulin therapy in order to retain metabolic control, followed by a repeat control imaging study. Since CT features of steatosis may be distinctively different from features of GlyH, low dose CT could be beneficial to differentiate between the two entities, but further studies are needed to validate this proposition (Glushko et al., 2018; Hsu et al., 2021). Furthermore, steatosis is often present in GlyH, possibly mitigating the discriminative power.

In case of remaining abnormalities or when in doubt, and after careful discussion with the patient, liver biopsy should be considered since this is still the golden standard to determine the cause of hepatopathy in T1D. Besides, when thinking of other concurrent liver conditions also leading to steatosis, e.g., alcoholic, auto-immune or viral hepatitis, a liver biopsy could be of added discriminative value.

### Conclusions in Key Points

NAFLD is very common in T2D, while its prevalence in T1D is still uncertain, although it seems more frequent than initially anticipated. The impact of NAFLD on the cardiometabolic risk profile of individuals merits further investigation.

T1D and T2D have a different physiopathology, however, there is increasing overlap between both entities. T1D patients are therefore not protected from the effects of overweight and/or insulin resistance. Patients with T1D and the metabolic syndrome pose the biggest risk of metabolic-associated hepatopathies and screening is recommended for these patients.

Fluctuations in glycemia and insulinemia are important factors in T1D-related liver steatosis. HbA1c is a good indicator of metabolic control, but does not evaluate large bidirectional excursions of glycemia. Intermittent and continuous glucose monitoring devices could aid in addressing this research question, since they can evaluate time spent in ideal range and glycemic variability.

Non-invasive diagnostics, including imaging studies and biomarkers, are largely unexplored to address NAFLD and the metabolic syndrome in individuals with T1D.

To date there is no licenced therapy for NAFLD or liver steatosis. Insight in the pathophysiology of diabetes-associated liver damage is needed to further expand therapeutic options.

GlyH is infrequent but likely underdiagnosed in T1D, is more benign than NAFLD, and both are associated with poor glycemic control. It is associated with extremely poor metabolic control, which is seen less in Western current practice, but it is not extinct. There are several clinical differences between both conditions that can aid the physician, but to date, liver biopsy remains the most accurate option to distinguish between both illnesses.

### Knowledge Gaps

Whether insulin resistance (and NAFLD) play a role in the pathogenesis of T1D, or *vice versa*, is unexplored. Longitudinal studies of new-onset patients with T1D are needed to evaluate the influence of metabolic disturbance on β-cell function and survival.

The possibility of selective insulin resistance at the level of the liver in patients with T1D is unaddressed.

Exact prevalence, incidence and (long-term) consequences of GlyH are unknown.

The burden of NAFLD and its hepatic and systemic consequences are largely unexplored in T1D.

The paradigm shift from NAFLD to MAFLD is not evaluated in T1D cohorts. Furthermore, no consequence is given to the interfering and potentially confounding role of GlyH in this shift, since imaging studies are sufficient according to the new definition to identify patients with MAFLD.

The effects of additive therapies focusing on components of the metabolic syndrome e.g., weight loss and insulin resistance are unexplored in T1D.

The role of glucometrics including time-in-range and glucose variability, in contrast to HbA1c, to evaluate metabolic burden including NAFLD in patients with T1D (and by extension T2D) needs to be explored.
